# Oral Postdialysis Cholecalciferol Supplementation in Patients on Maintenance Hemodialysis: A Dose-Response Approach

**DOI:** 10.1155/2014/597429

**Published:** 2014-01-21

**Authors:** Eric Descombes, Benoit Fellay, Ould Maouloud Hemett, Jean-Luc Magnin, Gilbert Fellay

**Affiliations:** ^1^Service of Nephrology, HFR Hôpital Cantonal, 1708 Fribourg, Switzerland; ^2^Central Laboratory, HFR Hôpital Cantonal, 1708 Fribourg, Switzerland

## Abstract

The aim of the present study was to evaluate the dose of postdialysis cholecalciferol needed to maintain the 25-hydroxyvitamin D [25(OH)D] levels in the optimal range of 75–150 nmol/L. Twenty-six patients who had low baseline 25(OH)D levels (mean 27.5 ± 14.9 nmol/L) were studied. The 25(OH)D levels were measured every 2 months for one year. During the first two months, all the patients received 2000 IU of cholecalciferol after each hemodialysis (=6000 IU/wk). Thereafter, the dose was individualized and adapted every 2 months by administering 1 to 6 cholecalciferol tablets (2000 IU each) per week (total weekly dose = 2000–12000 IU/wk). During cholecalciferol supplementation, the 25(OH)D concentrations rapidly increased from baseline to 140.1 ± 28.3 nmol/L at month 6 and 95.6 ± 20.9 nmol/L at month 12. At month twelve, 86% of the patients had 25(OH)D levels within the target range with a mean dose of 5917 ± 4106 IU/wk of cholecalciferol; however, the amount needed to maintain these levels varied widely from 0 (*n* = 2) to 12000 IU/wk (*n* = 5). In conclusion, postdialysis cholecalciferol prescription is quite effective in correcting vitamin D deficiency/insufficiency, but the amount of cholecalciferol needed to maintain the 25(OH)D levels within the optimal range over the long-term varies widely among patients and must be individualized.

## 1. Introduction

Recent important advances have been made in understanding vitamin D physiology, beyond its classic role in mineral and bone metabolism [1–11]. Indeed, recent studies have shown that several tissues, in addition to the kidneys, express the enzyme CYP27B1, which catalyzes the 1*α*-hydroxylation of 25(OH)D, and that the vitamin D receptor (VDR) is expressed ubiquitously [1–11]. It is now known that a conversion of 25(OH)D to 1*α*,25-dihydroxyvitamin D (calcitriol, the active form of vitamin D) occurs in several extrarenal cells and may be associated with significant biological roles beyond those traditionally attributed to vitamin D [1–11]. As a consequence, there has been a great deal of interest in the study of these nonclassical autocrine/intracrine effects of vitamin D during the past few years and a significant body of information in the medical literature has shown that vitamin D deficiency/insufficiency is associated with several abnormalities such as an increased risk of cardiovascular, musculoskeletal and autoimmune diseases, cancer, infections, diabetes, and mortality [1–6, 12–27]. As a result, the possible benefits of vitamin D supplementation in patients with low levels became the focus of interest of the scientific community and studies have even reported an improved survival in patients receiving cholecalciferol supplements [28, 29]. Accordingly, recent recommendations support the supplementation of vitamin D—with a dose equivalent to at least 800 IU per day—in a variety of clinical conditions, including chronic kidney disease (CKD) and, according to a majority of experts, the objective should be to achieve 25(OH)D concentrations of at least 75 nmol/L for an optimal health benefit [1, 6, 8, 30–39].

In patients who have CKD or who are on maintenance hemodialysis (HD) vitamin D deficiency/insufficiency is very widespread [9–11, 39–56]. Recent guidelines also recommend giving oral vitamin D supplementation to patients on maintenance hemodialysis who have low serum levels [36–39]. To accomplish this, the most recent KDIGO guidelines suggest using “treatment strategies recommended for the general population” [39]. In fact, several strategies of supplementation have been used or proposed in the literature for patients on maintenance HD and have included daily vitamin D supplementation to weekly or monthly high-dose prescriptions of either ergocalciferol or cholecalciferol, with doses ranging from 10000 IU/wk to 200000 IU/month [10, 44–55] or even 200000 IU/week [56]. However, data are still unavailable on regular post-HD vitamin D supplementation based on a dose-response approach.

Since the early 1990s, we have at our dialysis center prescribed a regular postdialysis oral supplementation of hydrosoluble vitamins with very satisfactory results [57–59]. This type of prescription makes it possible to achieve nearly 100% compliance to therapy and to reduce the number of tablets given to patients who already receive a high burden of oral medications [60]. It is well accepted by a majority of the patients and—in our experience—is cost-effective [58]. Thus, we were interested in adding cholecalciferol to the postdialysis multivitamin tablets which our patients already were receiving and in prospectively evaluating the dose of cholecalciferol needed to maintain the 25(OH)D serum levels in the optimal range of 75–150 nmol/L. Although there still is some debate concerning the target concentrations to be achieved, this range is the one considered to provide the optimal health benefit and optimal safety by many experts [1, 6, 8, 30–38] and is the one used at our institution.

## 2. Patients and Methods

For more than 10 years, a majority of the patients using the services of our dialysis center routinely receive—as do many Swiss HD patients—a post-HD supplement containing 5 hydrosoluble vitamins (*Dialvit*, Bichsel AG Interlaken, Switzerland). The composition of this multivitamin supplement is based on the results of several studies performed at our center [57–59] and is usually administered as two tablets after each dialysis session three times weekly. To correct vitamin D deficiency/insufficiency without increasing the tablet count [60], we therefore intended to prescribe these same tablets to which an additional 2000 IU of cholecalciferol had been added. These multivitamin tablets containing cholecalciferol (*Dialvit D*, Bichsel AG Interlaken, Switzerland) had already been used at another Swiss dialysis center and were well tolerated by the patients [61]. However, the tablets had been prescribed on a fixed schedule (2000 IU of cholecalciferol three times weekly), and some patients had very high 25(OH)D blood levels (personal communication). Thus, our objective was to individualize the dose of cholecalciferol supplementation by replacing some of the 6 standard tablets given each week with these cholecalciferol-enriched tablets. As can be seen in Table 1, this approach makes it is possible to administer weekly supplementation ranging from 2000 to 12000 IU per week. With this large range of different weekly doses, it is thus possible to accurately individualize the dose of the cholecalciferol supplement in order to achieve 25(OH)D levels within the target range of 75 to 150 nmol/L.

Consistent with the above discussion, we proposed to all of the patients at our center with low 25(OH)D levels (and normal calcium concentrations) who had not taken vitamin D supplementation previously and who were already receiving post-HD *Dialvit* that they replace some of the *Dialvit* tablets with these cholecalciferol-enriched ones in order to raise the vitamin D levels into the target range. Twenty-six patients agreed and thus received post-dialysis cholecalciferol supplementation. Their mean [±SD] age was 68 ± 9.8 years; 58% were males and all were Caucasian. The etiologies of renal failure were diabetic nephropathy (*n* = 7), glomerulonephritis (*n* = 7), ADPKD (*n* = 4), hypertension (*n* = 3), and miscellaneous (*n* = 5). The mean BMI of these 26 patients was 28.4 ± 5.1 kg/m^2^. On average, they have been on maintenance HD for 40 ± 28 months. They were dialyzed 3 times weekly with high-flux hemodialysis for an average dialysis time of 4 hours ± 15 minutes with a mean Kt/V of 1.41 ± 0.23. The calcium concentration of the dialysate was either 1.25 or 1.50 mmol/L. At baseline, the mean hemoglobin was 118.7 ± 10.8 g/L, the mean albumin 40.7 ± 3.2 g/L, and the mean CRP 7.5 ± 7.9 mg/L. The results of these 26 patients could be compared to those of twelve patients (mean age 63 ± 9.5 years, 75% males, 100% Caucasian) who were taking hydrosoluble multivitamin supplementation at home and who were not receiving vitamin D.

Overall, the patients had their 25(OH)D levels measured before dialysis at baseline and then once every two months with the *Roche vitamin D total* assay (Cobas 6000) for one year (from January to January). The accuracy of this automated immunoassay for 25(OH)D determinations in HD patients had been evaluated by our central laboratory in comparison with liquid chromatography-tandem mass spectrometry [62] and yielded satisfactory results [63]. The mean baseline 25(OH)D level of the 26 patients was 27.5 ± 14.9 nmol/L (range 5 to 57) and 50% of them had levels ≤25 nmol/L. During the first two months, all 26 patients received a supplement of 2000 IU of cholecalciferol ( = 6000 IU/wk) after each HD by replacing one of the two prescribed tablets of *Dialvit* with one that contained added cholecalciferol (*Dialvit D*, Bichsel AG Interlaken, Switzerland). After the first two months, patients still exhibiting 25(OH)D levels <75 nmol/L had their post-HD supplement increased to the maximum dose of 4000 IU/dialysis (2 tablets/HD = 12000 IU per week). After month 4, the dose of the cholecalciferol supplement was individualized and adapted every 2 months by giving 1 to 6 tablets of the cholecalciferol-enriched preparation on a weekly basis according to the modalities indicated in Table 1. The objective was to maintain the 25(OH)D levels within the target range of 75 to 150 nmol/L.

At baseline, all of the patients were receiving a phosphate-binder, 81% a calcium-based one—mostly calcium acetate (mean daily dose = 2 ± 1.5 g/day)—and 23% sevelamer. Sixty-nine percent were already receiving low-dose oral calcitriol (Rocaltrol, mainly given post-dialysis), 19% cinacalcet (Mimpara), and one patient intravenous paricalcitol (Zemplar). No patients had previously been given vitamin D supplements. During supplementation with cholecalciferol, the parameters of the mineral/bone metabolism were monitored at least once each month according to the standard protocol of our center. Ionized calcium, alkaline phosphatase, iPTH, and calcitriol (1–25(OH)_2_D) were measured at least once every 6 months; ionized calcium and iPTH were measured more frequently in patients receiving cinacalcet or if otherwise indicated. Note that during the one-year period the aforementioned treatments for the mineral/bones abnormalities were continued and adapted in the customary manner of our center, in general according to the most recent KDIGO guidelines [39]. It should also be noted that during the evaluation period the new guidelines for target iPTH levels were gradually introduced at our center. This resulted in a reduced use of PTH-lowering medications—that is, calcitriol and cinacalcet—and explains the fact that the iPTH concentrations tended to increase rather than to diminish in our patients, both in those receiving and those not receiving cholecalciferol supplementation.

Blood samples were drawn before the mid-week dialysis session and the following assays were performed according to the manufacturer's instructions: (1) Calcium Gen.2 (*System Roche/Hitachi cobas C 501*); (2) Parathormone-PTH intact (*System Roche/Hitachi cobas e 601*); (3) Alkaline Phosphatase acc. to IFCC Gen.2 (*System Roche/Hitachi cobas C 501*); (4) 25-Hydroxy vitamin D2 and D3 total (*System Roche/Hitachi cobas e 601*); (5) Calcitriol 1,25-Dihydroxy Vitamin D (kit *RIA, Immunodiagnostic system; reader:Wizard gamma Counter, PerkinElmer*).

The results are given as mean ± standard deviation. For continuous variables, the difference between two groups was assessed by the student's *t*-test and parameters with repeated measurements were compared with the one-way analysis of variance (ANOVA). All tests were two-sided and, since there were multiple comparisons, significance was deemed to exist for *P* < 0.01 (Bonferroni correction). Correlation coefficients were determined with the Pearson-Product Moment correlation method.

## 3. Results

**Figure 1:**
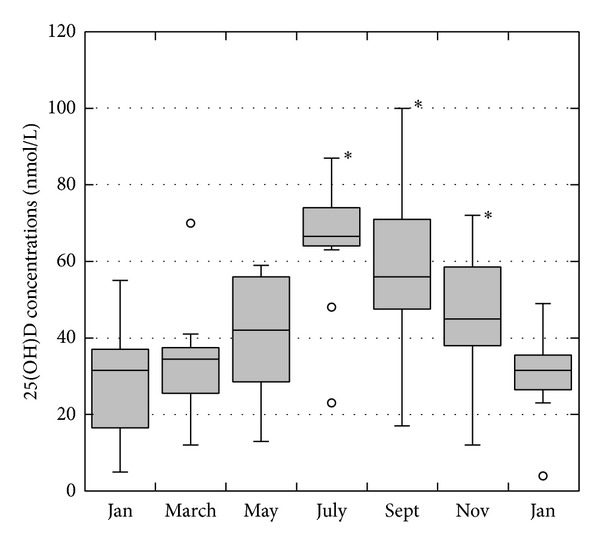
Boxplot showing the bimonthly 25(OH)D concentrations in patients not receiving vitamin D supplementation (*P < 0.001 compared to baseline).

In the 12 patients not receiving vitamin D, there was a significant seasonal increase of the 25(OH)D levels in summer: the levels increased from a baseline of 29.2 ± 15.6 nmol/L in January to 65.6 ± 17.0 nmol/L in July (*P* < 0.01) and then returned to 30.5 ± 10.8 nmol/L in January one year later.  Figure 1 shows boxplots for the bimonthly 25(OH)D concentrations in these patients. It is interesting to note that during the entire year, except for a few summer months, the majority of patients had persistently low vitamin D levels.  Table 2 also shows that, with the exception of the 25(OH)D levels, no significant changes in the other parameters of mineral/bone metabolism occurred during the observation period.

**Figure 2:**
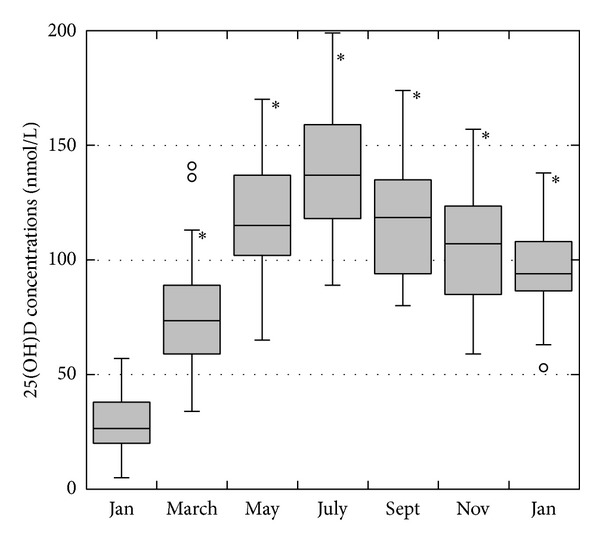
Boxplot showing the bimonthly 25(OH)D concentrations in patients receiving cholecalciferol supplementation (*P < 0.0001 compared to baseline).

In the supplemented patients, the cholecalciferol-enriched multivitamin tablets were well tolerated by all recipients and the development of the main parameters is summarized in Figure 2 and Table 2. During the correction phase, the 25(OH)D concentrations increased rapidly to levels significantly higher than those of the nonsupplemented patients, from 27.5 ± 14.9 nmol/L at baseline to 140.1 ± 28.3 nmol/L in July (month 6). Thereafter, in the maintenance phase, the serum levels stabilized progressively at 95.6 ± 20.9 nmol/L (month 12). Note that at month six 35% of the patients had 25(OH)D levels >150 nmol/L (but <200 nmol/L). This was probably due to the combination of a higher mean dose of cholecalciferol supplementation (7000 ± 3210 IU/wk) with the seasonal increase in vitamin D.

**Figure 3:**
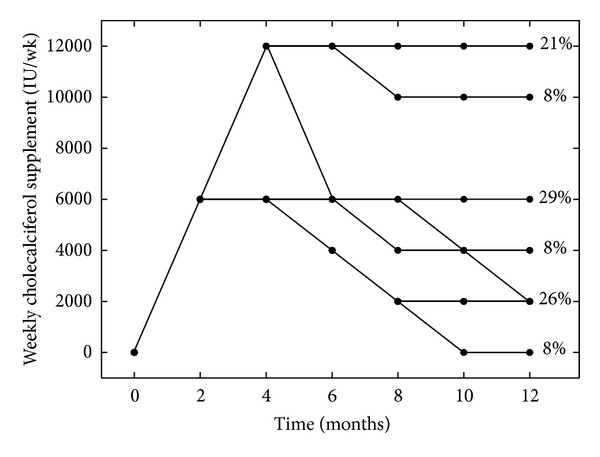
Development over time of the weekly dose of the cholecalciferol supplements and the respective percentages at month 12.

After a year of cholecalciferol supplementation (month 12), 86% of the patients had 25(OH)D levels within the target range (100% were between 50 and 150 nmol/L, Table 2) with average supplementation of *≈*6000 ± 4000 IU/week. However, the amount of cholecalciferol needed to maintain the 25(OH)D levels in the target range varied widely among the patients, from 0 (2 patients) to 12000 IU per week (5 patients): 42% of the patients required 4000 IU/wk of cholecalciferol or less and 29% required 8000 IU/wk or more (Table 2). Of the 5 patients receiving our maximum maintenance dose of cholecalciferol (12000 IU/wk), two still had 25(OH)D levels <75 nmol/L at month 12. It is interesting to note that, as shown in Figure 3, most of the changes in the prescribed cholecalciferol dose occurred during the first 6–8 months of supplementation. After month 8, the maintenance cholecalciferol dose prescribed did not change in any more patients, except for 3 (12%).

**Figure 4:**
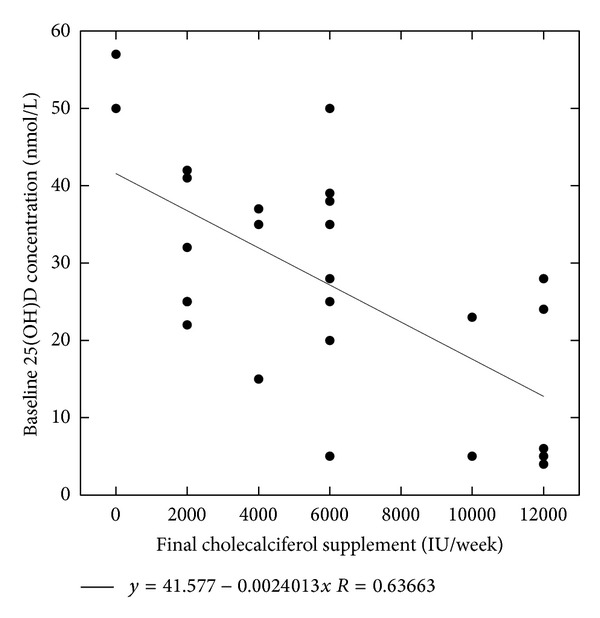
Correlation between the baseline 25(OH)D concentrations and the final maintenance dose of the cholecalciferol supplements (at month 12).

We analyzed the relationship between the baseline 25(OH)D concentrations (measured in January) and the final maintenance doses of the cholecalciferol supplement. It turned out that the patients needing cholecalciferol supplementation >6000 IU per week (mean 11430 ± 975 IU/wk) had mean baseline 25(OH)D concentrations significantly lower than those receiving 6000 IU/wk or less (mean 3790 ± 2200 IU/wk): 13.6 ± 10.8 versus 32.7 ± 12.9 nmol/L (*P* < 0.01).  Figure 4 actually shows that there was a negative correlation between the baseline 25(OH)D concentrations and the final maintenance dose of the cholecalciferol supplement (*r* = −0.637). This figure also shows that for patients having a baseline 25(OH)D concentration >25 nmol/L in January a post-dialysis supplement of 6000 IU of cholecalciferol per week or less was generally sufficient to achieve the target levels. On the other hand, more than 50% of those patients with baseline 25(OH)D levels ≤25 nmol/L required higher doses.

Concerning the parameters of mineral/bone metabolism, Table 2 shows that during cholecalciferol supplementation no significant changes occurred: the serum levels were comparable in supplemented and nonsupplemented patients (Table 2). This was also the case for the mean calcitriol (1–25(OH)_2_D) levels which remained essentially unchanged in both groups. During the observation period, 3 patients receiving both calcitriol and cholecalciferol and one patient not receiving cholecalciferol supplementation experienced transient episodes of mild asymptomatic hypercalcemia that rapidly regressed after the discontinuation of calcitriol.

## 4. Discussion

The data of the present study yield three main findings concerning vitamin D metabolism and post-dialysis cholecalciferol supplementation in patients on maintenance hemodialysis. First, in Swiss HD patients not receiving vitamin D supplementation, the 25(OH)D levels largely exhibit a seasonal variation. Second, regular post-dialysis oral cholecalciferol supplementation is quite effective in achieving 25(OH)D repletion in almost all patients. Third, the amount of oral post-dialysis cholecalciferol supplementation needed to maintain the vitamin D levels within the target range varies widely among patients and is related to the baseline 25(OH)D concentrations.

In nonsupplemented HD patients, the results clearly show a seasonal rhythm of the 25(OH)D levels, with an important peak occurring in summer when the mean levels are more than twice those observed in winter. This circannual rhythm and its magnitude correspond to those reported for the general Swiss population, which exhibits its highest 25(OH)D levels between the months of July and September [64, 65] (Note: the latitude of Switzerland is 46°-47° north). Similar results also have been reported in other countries with seasonal peaks of course largely being influenced by the intensity of sun exposure both in individuals with normal renal function and patients with stage 5 CKD [1, 2, 8, 66–69]. As the data suggest that patients on maintenance HD exhibit a seasonal behavior of vitamin D that is comparable to that of the general population, this implies that the prevalence of vitamin D deficiency/insufficiency in HD patients will largely vary according to the time of the year. From a practical point of view, these findings have two clinical consequences. First, most of recent recommendations propose screening vitamin D levels at “baseline” in patients with CKD and then supplementing individuals with low levels. However, depending on the season in which the screening is performed, the results can be expected to differ considerably [64, 66]. As a consequence, we consider that the results of a single 25(OH)D measurement should always be interpreted in light of the season in which the measurement is performed and that a single measurement generally is insufficient to accurately evaluate the vitamin D status of a specific patient on an annual basis [66]. Second, these seasonal changes also will influence the 25(OH)D levels in patients receiving vitamin D supplementation. Therefore, their levels can be expected to be higher in summer and lower in winter, as recently reported by a Spanish group [54].

For many years at our dialysis center, we used to prescribe regular post-dialysis oral supplementation of hydrosoluble vitamins with quite satisfactory results [57–59]. As mentioned in the introduction, this type of prescription has many advantages. The main advantage is achieving a nearly 100% compliance rate to the administered treatment. This is usually not the case for daily prescriptions which, according to the literature, are associated with a compliance rate that generally does not exceed 70% [58]. It is for this reason that we considered prescribing vitamin D supplementation on a post-dialysis basis by using the same tablets that contained the hydrosoluble vitamins and achieved quite satisfactorily results. Our results clearly show that regular post-dialysis oral supplementation with cholecalciferol is very effective. It achieves 25(OH)D repletion in almost all of the patients with a dose of cholecalciferol averaging *≈*6000 IU/week. This dose roughly corresponds to 850 units of cholecalciferol per day (or *≈*25000 units/month)—a dose which is very close to the supplement that is often recommended for the general adult population [1, 6, 8, 30–38] but is much lower than the weekly or monthly high-dose supplements prescribed for HD patients in several previous studies—up to 200000 IU/month [10] or even 200000 IU/week [56]. This dose is also lower than the doses proposed by the KDOKI guidelines for initial vitamin D supplementation in patients with stage 3 and 4 CKD exhibiting vitamin D deficiency—that is, 50000 IU/wk of ergocalciferol for 4 to 12 weeks. It remains unclear why there is such great variance in the recommended doses of vitamin D supplements and additional studies should investigate this point. Interestingly, a previous study has shown that the response to an equivalent cumulative dose of cholecalciferol is the same with daily, weekly or monthly dosing frequencies [70]. Thus, in our opinion, it cannot be ruled out that these discrepancies may at least partially be related to differences in the bioavailability of the large variety of vitamin D compounds found throughout the world. Also worth noting is the fact that the literature contains some discussion about the respective efficacy of ergocalciferol supplementation compared to that using cholecalciferol [8, 11, 71–74]. Lastly, it should also be noted that a great variation does exist in the accuracy of the available automated immunoassays for 25(OH)D determinations, particularly in patients on dialysis [63, 75, 76]: this may results in either an overestimation or an underestimation of the 25(OH)D serum levels and may explain some of the apparent discrepancies among the results reported in the literature.

In our patients, the amount of the oral post-dialysis cholecalciferol supplement needed to maintain the 25(OH)D levels within the target range varied widely. While more than one-third of our patients required cholecalciferol supplementation of less than 6000 IU/wk (4000 or 2000 IU/wk) to achieve vitamin D repletion, nearly one-third required much higher doses, namely, up to 12000 IU/wk (*≈*50000 IU/month). This heterogeneous response suggests that patients with advanced CKD also exhibit individual differences in vitamin D metabolism and respond differently to vitamin D supplementation. This is already known from previous studies on healthy subjects. In this regard, Binkley et al. noted that “as vitamin D is in essence an endogenously produced hormone, it is not surprising that between-individual variability and regulation would exist” [8]. These authors reported that the human skin is able to regulate cholecalciferol production and that there are also differences between individuals in CYP24A1 capacity during vitamin D degradation [77]. Notably, studies from sunny countries also support between-individual differences in vitamin D metabolism despite abundant sun exposure [78, 79].

Our results also show that there is an inverse correlation between the baseline 25(OH)D concentrations and the maintenance dose of cholecalciferol needed to maintain serum levels within the target range. This is consistent with previous studies which have shown that using fixed doses of vitamin D in patients with lower baseline levels results in lower steady state concentrations under supplementation and requires higher doses of the supplement to further increase the patient serum 25(OH)D levels [8, 11, 14, 31, 33, 38, 48, 70]. Holick and Chen emphasized that the baseline 25(OH)D concentration is an important factor in the individual response to vitamin D supplementation [2]. It has been considered that a daily supplement of 400 IU of vitamin D2/D3 is expected to induce an increase in the 25(OH)D serum levels of 10 nmol/L in healthy subjects [2, 8, 34]. Concerning dialysis patients, Jean et al. in their first study used different doses of cholecalciferol ranging from 400 to 1200 IU per day that were prescribed according to patient baseline 25(OH)D levels [51]. They reported that after six months 30% of their patients had 25(OH)D levels >150 nmol/L (i.e., the upper limit of their target levels), but they did not analyze the development of the dose-response time course in any greater detail [51]. In patients with stage 3 and 4 CKD, the KDOQI guidelines also recommend different doses of vitamin D supplementation according to the severity of the initial vitamin D deficiency/insufficiency [39]. Concerning regular post-dialysis cholecalciferol prescription and based on the results of the present study, we propose initiating supplementation with a fixed dose of 2000 IU three times weekly. According to our results, we expect that this dose will suffice or even be too high in patients with baseline 25(OH)D levels >25 nmol/L (as measured during winter). However, it should be insufficient for more than the one-half of those with baseline levels ≤25 nmol/L. Thus, we are of the opinion that the 25(OH)D levels should be monitored at 2-3 and then at 4–6 months after the initiation of vitamin D supplementation in order to determine and individualize the maintenance dose. Thereafter, since the maintenance dose usually remains steady in individual patients and considering the relatively high cost of 25(OH)D determinations compared to the cost of the vitamin D itself, further monitoring should be considered either on an individual basis or once/twice each year. It should be noted that presently, based on our data and the data reported by González-Parra et al. [54], we would look for annual target 25(OH)D levels of 100–125 nmol/L on average, in order to anticipate the expected seasonal fluctuations of vitamin D levels.

Concerning the parameters of mineral/bone metabolism, we did not observe significant changes of the calcium and phosphate mean levels under cholecalciferol supplementation and this is consistent with the data available in the literature [10, 33, 36]. Prior studies actually reported that supplementation with ergocalciferol or cholecalciferol has no significant effects on the phosphate levels and may be associated with hypercalcemia in only 3% of the patients [10]. In our cohort, mild transient episodes of hypercalcemia occurred in 3 patients who were at the same time also receiving calcitriol. This is in agreement with the data reported in healthy volunteers by Heaney et al. showing that the calcium homeostasis is already influenced by low doses of calcitriol but not by cholecalciferol supplementation unless very high doses are prescribed, that is, 50000 IU/day [80]. Jean et al. reported that cholecalciferol prescription is associated with a reduction of the iPTH levels [51, 81]. This was not the case in our cohort for the reasons specified in the method section (i.e., a reduced use of PTH-lowering medications during the studied period due to changes in KDIGO guidelines), and therefore this point cannot be analyzed any further in our patients.

## 5. Conclusions

The results of the present study show that in patients on maintenance hemodialysis with low vitamin D levels, regular oral post-dialysis cholecalciferol supplementation (prescribed by us as multivitamin tablets that also contained hydrosoluble vitamins) is quite effective in achieving 25(OH)D repletion in almost all patients. The cholecalciferol dose needed to achieve vitamin repletion was on average *≈*6000 IU/week—that is, 2000 IU three times weekly—but the response to vitamin D supplementation was highly variable among patients and thus the maintenance dose of vitamin D to be prescribed over the long-term must be individualized.

## Figures and Tables

**Table 1:** Range of the post-dialysis dose of cholecalciferol supplements according to tablet prescription.

Cholecalciferol	Multivitamins*	Multivitamins + D3**	Approximate VitD	Approximate VitD
Weekly dose (IU)	tbls/week	tbls/week	Daily dose (*≈*IU)	Monthly dose (*≈*IU)
0	6	0	0	0
2000	5	1	285	8000
4000	4	2	570	16000
6000	3	3	855	26000
8000	2	4	1140	34000
10000	1	5	1425	42000
12000	0	6	1710	52000

*Tablets containing 5 hydrosoluble vitamins (*Dialvit*).

**The same tablets with added 2000 IU of cholecalciferol/tablet (*Dialvit* D).

**Table 2:** Bimonthly development of the biological parameters, as well as the dose of the post-dialysis cholecalciferol supplements, and comparison with the results in patients not receiving vitamin D supplementation.

Month	Winter			Summer			Winter	ANOVA
	Jan	March	May	July	Sept	Nov	Jan	
*n *	26	26	26	26	24°	24	24	
Mean 25(OH)D concentration (nmol/L)	27.5 ± 14.9	76.1 ± 28.1*	119.8 ± 27.7*	140.0 ± 28.3*	117.4 ± 25.9*	106.2 ± 23.6*	93.9 ± 19.6*	*P* < 0.0001
Range of 25(OH)D concentrations (nmol/L)	5–57	34–140	65–170	89–199	80–174	59–157	53–138	
Mean weekly dose of cholecalciferol (IU/week)	0	6000	7846 ± 2824	7000 ± 3212	6250 ± 3791	6000 ± 4043	5917 ± 4106	
Range of the weekly dose of cholecalciferol (IU)	0	all 6000	6000 or 12000	4000–12000	2000–12000	0–12000	0–12000	
Distribution of the 25(OH)D concentrations								
>150 nmol/L	0	0	30%	35%	8%	4%	0	
75.1–150 nmol/L	0	69%	76%	65%	92%	92%	84%	
50.1–75 nmol/L	4%	18%	4%	0	0	4%	16%	
25.1–50 nmol/L	46%	13%	0	0	0	0	0	
0–25 nmol/L	50%	0	0	0	0	0	0	
Distribution of the weekly doses of the cholecalciferol supplement								
8000–12000 (IU/week)	0	0	31%	27%	29%	29%	29%	
6000 (IU/week)	0	100%	69%	42%	34%	29%	29%	
0–4000 (IU/week)	0	0	0	31%	37%	42%	42%	
Parameters of bone metabolism								
Calcium (mmol/L)	2.29 ± 0.16	2.36 ± 0.18	2.36 ± 0.19	2.37 ± 0.16	2.37 ± 0.16	2.34 ± 0.17	2.32 ± 0.15	NS
Phosphate (mmol/L)	1.54 ± 0.26	1.75 ± 0.43	1.67 ± 0.43	1.66 ± 0.34	1.59 ± 0.42	1.73 ± 0.40	1.72 ± 0.37	NS
Alkaline phosphatase (U/L)	107.0 ± 51.1			97.0 ± 43.4			101.8 ± 45.7	NS
iPTH (ng/L)	252.1 ± 141.6			263.1 ± 159.0			388.2 ± 244.7	NS
Mean 1–25(OH)_2_D concentration (pmol/L)	32.9 ± 15.1			32.5 ± 23.1			30.1 ± 11.0	NS
Unsupplemented patients (*n* = 12)								
Mean 25(OH)D concentration (nmol/L)	29.2 ± 15.6	33.1 ± 14.6	40.3 ± 15.4	65.6 ± 17.1	59.7 ± 22.0	46.5 ± 17.7	30.5 ± 10.8	*P* < 0.0001
Calcium (mmol/L)	2.30 ± 0.17	2.30 ± 0.13	2.27 ± 0.17	2.27 ± 0.16	2.35 ± 0.33	2.31 ± 0.12	2.32 ± 0.17	NS
Phosphate (mmol/L)	1.76 ± 0.46	1.64 ± 0.45	1.66 ± 0.51	1.58 ± 0.65	1.59 ± 0.55	1.46 ± 0.45	1.65 ± 0.38	NS
Alkaline phosphatase (U/L)	101.3 ± 46.6			97.9 ± 43.5			107.9 ± 48.5	NS
iPTH (ng/L)	263.7 ± 184.7			323.5 ± 228.8			332.5 ± 272.0	NS
Mean 1–25(OH)_2_D concentration (pmol/L)	29.3 ± 15.7			35.3 ± 32.9			30.2 ± 18.8	NS

*P < 0.01 compared to baseline and to patients without vitamin D supplementation.

°Between months 6 and 8, one patient has undergone kidney transplantation and another died.
